# The Effect of High-Dose Vitamin C on Biochemical Markers of Myocardial Injury in Coronary Artery Bypass Surgery

**DOI:** 10.21470/1678-9741-2018-0312

**Published:** 2019

**Authors:** Nafiseh Emadi, Mohammad Hasan Nemati, Mohammad Ghorbani, Elahe Allahyari

**Affiliations:** 1 Blood Circulation Technology, Shiraz University of Medical Sciences, Shiraz, Iran.; 2 Department of Heart Surgery, Shiraz University of Medical Sciences, Shiraz, Iran.; 3 Anesthesiology Research Center, Shiraz University of Medical Sciences, Shiraz, Iran.; 4 Department of Public Health, Torbat Heydarieh University of Medical Sciences, Torbat Heydarieh, Iran.; 5 Department of Anesthesiology, Shiraz University of Medical Sciences, Shiraz, Iran.

**Keywords:** Cardioplegic Solutions, Stroke Volume, Coronary Artery Bypass, Ventricular Function, Arrhythmias, Cardiac, Biomarkers, Vitamins, Ascorbic Acid

## Abstract

**Objective:**

To evaluate the effect of high-dose vitamin C on cardiac reperfusion injury and plasma levels of creatine kinase-muscle/brain (CK-MB), troponin I, and lactate dehydrogenase (LDH) in patients undergoing coronary artery bypass grafting (CABG).

**Methods:**

This is a double-blind randomized clinical trial study. Fifty patients (50-80 years old) who had CABG surgery were selected. The intervention group received 5 g of intravenous vitamin C before anesthesia induction and 5 g of vitamin C in cardioplegic solution. The control group received the same amount of placebo (normal saline). Arterial blood samples were taken to determine the serum levels of CK-MB, troponin I, and LDH enzymes. Left ventricular ejection fraction was measured and hemodynamic parameters were recorded at intervals.

**Results:**

High doses of vitamin C in the treatment group led to improvement of ventricular function (ejection fraction [EF]) and low Intensive Care Unit (ICU) stay. The cardiac enzymes level in the vitamin C group was lower than in the control group. These changes were not significant between the groups in different time intervals (anesthesia induction, end of bypass, 6 h after surgery, and 24 h after surgery) for CK-MB, LDH, and troponin I. Hemodynamic parameters, hematocrit, potassium, urinary output, blood transfusion, arrhythmia, and inotropic support showed no significant difference between the groups.

**Conclusion:**

Vitamin C has significantly improved the patients’ ventricular function (EF) 72 h after surgery and reduced the length of ICU stay. No significant changes in cardiac biomarkers, including CK-MB, troponin I, and LDH, were seen over time in each group.

**IRCT code:**

IRCT2016053019470N33

**Table:** 

Abbreviations, acronyms & symbols				
ACT	= Activated clotting time		iNOS	= Inducible nitric oxide synthetase
AF	= Atrial fibrillation		IQR	= Interquartile range ok
AV-block	= Atrioventricular block		K	= Potassium
BSA	= Body surface area		LDH	= Lactate dehydrogenase
CABG	= Coronary artery bypass grafting		LIMA	= Left internal mammary artery
CK-MB	= Creatine kinase-muscle/brain		LVEF	= Left ventricular ejection fraction
CPB	= Cardiopulmonary bypass		MI	= Myocardial injury
CVA	= Cerebrovascular accident		nNOS	= Neuronal nitric oxide synthetase
DNA	= Deoxyribonucleic acid		NO	= Nitric oxide
EF	= Ejection fraction		PVC	= Premature ventricular contraction
HB	= Hemoglobin		SD	= Standard deviation
HCT	= Hematocrit		SPSS	= Statistical Package for the Social Sciences
Hlp	= Hyperlipidemia		TTE	= Transthoracic echocardiography
HTN	= Hypertension		V-tach	= Ventricular tachycardia
I/R	= Ischemia/Reperfusion		VF	= Ventricular fibrillation
ICU	= Intensive Care Unit			

## INTRODUCTION

The cardiopulmonary bypass (CPB) pump, which is used for coronary artery bypass grafting (CABG) surgery, can cause inflammatory reactions^[1]^. The harmful effect of CBP can be produced by free oxygen radicals^[2]^. During ischemia and reperfusion, the free radicals of oxygen affect the mitochondria, sarcoplasm, vascular endothelial cells, adenosine, and nitric oxide (NO), causing an undesirable effect. This effect increases the damage to the myocardium by arrhythmia, impaired contractions, and necrosis^[3]^. The systemic release of free radicals during CPB disrupts the reperfusion process of the ischemic heart and causes a secondary damage to the myocardium^[2]^.

On the other hand, the production of free oxygen radicals leads to the use of main antioxidants. The plasma concentration of vitamin C decreases by approximately 70% in all subjects under CPB within 24 hours after surgery, and this decrease persists in most patients for up to two weeks. Therefore, the serious reduction of vitamin C leads to destruction of the body’s defense against activated oxygen species produced during cardiac surgery^[4]^.

Studies have shown that the ascorbic acid acts as a free radical scavenger to reduce the lipid peroxidation in the cell membrane and effectively protects from myocardial damage against ischemic reperfusion, by removing the free radicals during and after surgery^[5,6]^.

Myocardial injury (MI) and myocardial infarction during the surgery are determined by measuring serum myocardial necrosis indices, such as creatine kinase-muscle/brain (CK-MB), troponin I, and lactate dehydrogenase (LDH). Cell membrane damage causes these cardiac markers to spread to the bloodstream^[7-9]^.

Regarding the mentioned issues, free radicals of oxygen are considered as the main cause of myocardial reperfusion ischemic injury in patients under CPB, which increases the myocardial damage, by arrhythmias, impaired contractions, and necrosis. Studies have been conducted to find and remove free radicals and protect the myocardium, including strengthening the antioxidant defense, which may help to protect tissues from reperfusion damage in surgical procedures under CPB. Vitamin C reduces free radicals, as an antioxidant, and may play an important role in reducing cardiac enzymes and postoperative complications. Our main objective in this study was to evaluate the effect of high doses of vitamin C on plasma levels of CK-MB, troponin I, and LDH (after CABG) and the cardiac protective effect of vitamin C. Also, the incidence of arrhythmia and the patients’ hemodynamic symptoms have been investigated.

## METHODS

This is a double-blind randomized clinical trial study. The study was approved by the Ethics Committee of the Shiraz University of Medical Sciences (No. 10263). Fifty patients, males and females, with the age range of 50-80 years and who had CABG surgery were selected using a simple sampling method for the study. The inclusion criteria were: patients who referred to Shiraz hospitals for undergoing CABG and who signed the written consent for the participation in the study. The exclusion criteria were: history of arrhythmias, ejection fraction (EF) < 30%, severe renal or hepatic failure, permanent or temporary pacemaker, treatment with antiarrhythmic drugs and digoxin, presence of a degree of atrioventricular block (AV-block) or bradycardia with a rate of < 50 per minute, history of recent myocardial infarction (less than a month), high initial troponin I level, and history of redo or complex (valve replacement + CABG) surgery. Left ventricular ejection fraction (LVEF) was measured by transthoracic echocardiography (TTE). Sample selection was done using a block randomization method. A demographic information form was completed for the patient.

Patients in group A received 5 g of intravenous vitamin C before induction of anesthesia and 5 g of vitamin C in the cardioplegic solution. Patients in group B, as control group, were given the same amount of placebo (normal saline). Arterial blood samples were taken at the following intervals to determine the serum levels of CK-MB, troponin I, and LDH: during induction of anesthesia, at the end of CABG, six hours after surgery, and 24 hours after surgery. In all patients, blood samples were taken before the injection of vitamin C or placebo and considered as baseline. Hemodynamics of patients, including systolic and diastolic pressures, mean arterial pressure, and heart rate, were recorded at specified intervals.

The method of anesthesia was the same in all patients. The induction of general anesthesia was done by midazolam (0.1-0.2 mg/kg), fentanyl (5-10 µg/kg), sodium thiopental (1-3 mg/kg), and pancuronium bromide (0.1 mg/kg) and it was maintained with propofol (50-100 µg/kg/min), remifentanil (0.1-0.2 µg/kg/min), and oxygen and nitrous oxide (50%/50%). After the sternotomy and separation of the graft (right or left internal mammary artery [LIMA], saphenous vein, and radial artery), following the administration of heparin, at a dose of 300 u/kg and achieving activated clotting time (ACT) up to 400 seconds, the aortic and right atrium cannulation was performed by the surgeon and CPB pump was used. After the patient's aortic clamping, a cold blood cardioplegic solution containing 5 g of vitamin C or placebo was injected, respectively, into group A and group B patients, through a cardioplegia cannula and using antegrade techniques. The cardioplegia used was the same in all patients, with the combination of blood and hypothermic crystalloid, and it was repeated every 20 minutes. At the same time, patients were cooled down to 32-34 °C. The surgical technique was the same in all patients and it was performed by the same surgeon. In all cases, LIMA and saphenous vein were used in CABG.

Anastomosis of the distal grafts (on the heart) was performed by the surgeon in patients in cardiac arrest. According to the surgeon opinion, the patients started to warm up, and after aortic declamping, any arrhythmia, such as irregular rhythm and rate (premature ventricular contracture, premature atrial contracture, ventricular tachycardia, atrial or ventricular fibrillation, asystole, bundle branch blocks), the time, and the method of intervention for the treatment of arrhythmia (lidocaine and amiodarone injection, shock) were recorded in the checklist. The rate of reception and the number of inotropic drugs prescribed for heart rehabilitation were also recorded. After proximal anastomosis on the aorta, the end of bypass is announced. Separation of patients from the CPB was based on the standard protocol, including the appropriate left ventricular filling pressure and proper hemodynamic using liquid and vasoactive drugs administration. Inotropic drugs were administered based on hemodynamic and underlying conditions of the patient. Epinephrine and dopamine were the first line inotropes to support the heart. At the end, after the patient's separation from the CPB pump, for heparin reversal, protamine sulfate was given to patients at a rate of 50 mg per 5000 units of heparin and ACT was checked again. After insertion of the chest tube and examination of the patient in terms of hemorrhage and hemostasis, in sternum, subcutaneous tissue, and sutured skin, patients were transferred to the Intensive Care Unit (ICU) and, after stable hemodynamic conditions, isolated from the ventilator in the ICU. The level of biochemical markers and the hemodynamic changes in the patient, the type and treatment of arrhythmia, and the time and number of inotropes were also recorded at six hours after surgery and 24 hours after surgery.

### Statistical Analysis

Data was analyzed using the Statistical Package for the Social Sciences (SPSS) software version 24. Qualitative variables were represented by number and percentage. Quantitative variables with normal distribution were represented with mean and standard deviation (mean ± SD), and quantitative variables with abnormal distribution with median and interquartile range (median [IQR]). To test the normality, Kolmogorov-Smirnov test was used. For comparison of the variables between the two groups according to the need, Student t-test, Mann-Whitney U test, and chi-square test and repeated measure were used. The values are significant when *P*<0.05.

## RESULTS

Table 1 shows the patients’ demographic data in the two groups. It indicates that the conditions of the patients in both groups were the same. The patients' information during the surgery is shown in Table 2.

**Table 1:** Patients' demographic data.

Variable	Vitamin C group	Control group	*P*-value
Age (years) (mean ± SD)	60±6.62	63.64±8.26	0.092
Gender (male/female) (%)	72/28	56/44	0.293
Height (cm) (mean ± SD)	168.52±6.18	167.24±7.17	0.50
Weight (kg) (mean ± SD)	74.68±8.60	74.32±13.64	0.91
BSA (m^2^) (mean ± SD)	1.82±0.11	1.82±0.18	0.90
Pre-operation EF (%) (mean ± SD)	56.29±6.29	56.50±6.12	0.96
Systolic pressure (mmHg) (mean ± SD)	134.76±33.8	144.92±29.07	0.42
Diastolic pressure (mmHg) (mean ± SD)	79.56±17.01	80.92±14.13	0.48
HCT (mean ± SD)	39.24±6.16	39.66±9.49	0.78
K (mEq/L) (mean ± SD)	3.11±0.44	3.05±0.43	0.18
HB (mean ± SD)	13.62±2.27	13.65±3.48	0.78
Diabetes (%)	32	60	0.35
HTN (%)	56	72	0.23
Hlp (%)	56	64	0.56
MI (%)	8	0	0.14
CVA (%)	4	8	0.99

BSA=body surface area; CVA=cerebrovascular accident; EF=ejection fraction; HB=hemoglobin; HCT=hematocrit; Hlp=hyperlipidemia; HTN=hypertension; K=potassium; MI=myocardial injury; SD=standard deviation

**Table 2:** Patients' information during the surgery.

Variable	Vitamin C group	Control group	*P*-value
Number of grafts	4.04±0.61	4.16±0.74	0.38
Aortic clamping time (min)	28.20±4.23	31.32±8.10	0.09
CPB time (min)	50.32±5.93	53.88±12.35	0.20

CPB=cardiopulmonary bypass

The levels of CK-MB, LDH, and troponin I in four times (anesthesia induction, end of bypass, six hours after surgery, and 24 hours after surgery) were measured and recorded. The values of each of the three biochemical markers at all times in the control group were greater than those of the treatment group. Data analysis was done in two ways: (1) comparison of changes in biochemical markers over time in each group and (2) comparing biochemical markers changes between the two groups in general and then at each time.

In the comparison of CK-MB enzymes over time in each group, the CK-MB levels from the first to the second time showed a sharp increase in both groups, there was a slow decrease from the third to the fourth time in the treatment group, whereas in the control group, after a slow decrease, the enzyme levels increased again. These changes were significant over time in each group (*P*=0.0001). In comparing the two times in each group, the first time showed a significant difference from the three other times (*P*=0.0001) and it indicates a significant increase in the enzyme during and after surgery, but the second, third, and fourth times have only significant difference from the first time, but not from other times (*P*=1). The comparison of CK-MB enzymes changes between the two groups reveals that the overall comparison between the two groups did not show statistically significant differences (*P*=0.27). Therefore, the enzyme values were evaluated in each time in two groups and there was no significant difference between the two groups (*P*>0.05).

In the comparison of troponin I changes over time within the groups, the treatment group was steadily increasing until the second time, and thereafter until the fourth time, there is a slow increase in the amount of troponin I. But in the control group, there is a rapid and steady increase in all times. These changes are significant in each group (*P*<0.05). In comparing two different times in each group, troponin I changes were significant between the first time and the second and third times (*P*=0.0001) and the fourth time (*P*=0.001), therefore there is a significant increase in the level of troponin I during and after surgery. The second, third, and fourth times showed a significant difference only from the first; no significant difference was showed from other times (*P*>0.05). The comparison of troponin I changes between the groups showed no significant difference (*P*=0.35). Also, the comparison of troponin I values in each of the four study times didn’t show significant differences between the two groups (*P*>0.05).

In the comparison of LDH enzymes over time in each group, from the first to the second time LDH was slowly increased in both groups and from the second to the third time, there was a great and fast increase and the increase in the control group was faster and bigger than in the treatment group. From the third to the fourth time, both groups had the same increase. Similarly to other enzymes, changes over time were significant (*P*<0.05). Comparison of two times in each group showed that the first time had a relatively significant relationship with the second time (*P*=0.057). And with the third and fourth times, it was completely significant (*P*=0.0001). The third and fourth times didn’t show significant changes (*P*=0.87). The comparison of LDH enzyme changes between the two groups showed no significant statistical difference (*P*=0.09) and also the amount of LDH enzyme was not statistically different at any time between two groups (*P*>0.05) (Table 3). Table 4 shows the patients' information during and after the surgery in the two groups.

**Table 3:** Patients' levels of CK-MB, LDH, and troponin I in four times.

	Anesthesia induction	End of bypass	6 h after surgery	24 h after surgery	*P*-value
**CK-MB**					
Vitamin C group	16.28±9.69	43.96±14.72	39.72±18.71	40.36±22.79	0.755
Control group	14.57±7.40	47.88±15.52	44.84±28.15	53.80±47.8	0.000
*P*-value	0.47	0.363	0.453	0.211	
**Troponin I**					
Vitamin C group	0.104±0.275	0.657±0.708	0.701±0.724	0.811±0.738	0.000
Control group	0.167±0.329	0.559±0.574	0.888±1.01	1.17±1.92	0.000
*P*-value	0.468	0.594	0.457	0.387	
**LDH**					
Vitamin C group	217.68±49.17	254.88±75.92	365.64±96.93	419.92±195.38	0.001
Control group	265.28±88.53	277.76±90. 57	442.88±173.66	487.76±337.10	0.16
*P*-value	0.052	0.338	0.06	0.388	

CK-MB=creatine kinase-muscle/brain; LDH=lactate dehydrogenase

**Table 4:** Patients' information during and after the surgery.

	Vitamin C group	Control group	*P*-value
Use of packed cell (mean ± SD)			
In operation	0.12±0.33	0.24±0.43	0.27
After operation	1.80±0.76	1.84±0.89	0.82
Inotrope (mean ± SD)			
Time (h)	4.04±5.41	2.32±5.18	0.15
Types (No) (%)			0.68
No type	9 (36)	12 (48)	
One type	15 (60)	12 (48)	
Two types	1 (4)	1 (4)	
EF (mean ± SD)			
After operation (%)	56.80±4.05	51.80±6.59	0.002*
EF changes(%)	0.80±6.27	-4.2±5.53	0.009*
Intubation time (h) (mean ± SD)	10.76±2.14	11.72±2.57	0.25
ICU stay (days) (mean ± SD)	1.22±0.27	1.58±1.18	0.012*
Hospital stay (days) (mean ± SD)	4.12 ± 0.33	5.16 ± 3.44	0.15

* Significant increase of EF between groups (*P*-value < 0/05)

EF=ejection fraction; ICU=intensive care unit; SD=standard deviation

EF was measured in all patients 72 hours after the operation by TTE. The comparison between the two groups showed that the percentage of EF in the control group, comparing with the treatment group, decreased, and this difference was statistically significant (*P*=0.002). Also, the EF changes variable, which indicates changes in EF from preoperative time to 72 hours after surgery, was compared in both groups. The results indicate that the EF of the treatment group experienced an increase of 0.8% and the control group experienced a decrease of 4.2% (*P*=0.009). These results show statistically significant differences in EF changes during 72 hours after operation between the two groups (Table 4).

The length of ICU stay in both groups was compared. The results showed that the patients in the treatment group were hospitalized for a shorter time in the ICU than those in the control group, which was statistically significant (*P*=0.012) (Table 4).

The urinary output of both groups of patients was measured in three times, before the onset of bypass (time 1), at the end of bypass (time 2), and 24 hours after surgery (time 3). The results show that the amount of urine output in patients was increased at the end of the pump until 24 hours after the surgery, and these changes were the same between the two groups. Although changes in time are significant, regardless of being in the treatment or control group (*P*=0.0001), there are no significant differences between the two groups (*P*=0.34).

Plasma levels of hemoglobin and potassium were measured at four-time intervals: anesthesia induction time, bypass termination, six hours after surgery, and 24 hours after surgery. The results did not show any significant difference between the treatment and control groups. Hemoglobin levels from the first to the second time, the end of bypass, showed a sharp decline, but increased in 24 hours after surgery in both groups. But these changes are not statistically significant between the two groups (*P*=0.89). Blood potassium levels in both groups increased by up to six hours postoperatively and decreased in 24 hours after surgery, and although these changes in all patients were meaningful over time (*P*=0.0001), there was no significant difference between the two groups (*P*=0.85).

Comparison of hemodynamic parameters, including systolic and diastolic blood pressures and heart rate, in both groups in the four mentioned times showed that until the third time (six hours after surgery), a decrease in blood pressure was observed, and thereafter a relative increase was detected. These changes are significant over time, but they are not statistically different between the two groups (*P*=0.58, *P*=0.91). Also, the heart rate of patients in the control group was lower than of those in the treatment group, and patients in both groups had the highest and the lowest heart rate in six and 24 hours after surgery, respectively. These changes were significant over time (*P*=0.004), but no significant differences were observed between two groups (*P*=0.56).

Table 5 shows the effect of vitamin C on the incidence of cardiac arrhythmias in five-time intervals, including: anesthesia induction, end of bypass, six hours after surgery, 24 hours after surgery, and at aortic declamping.

**Table 5:** The effect of vitamin C on the incidence of cardiac arrhythmias and its treatment in five-time intervals.

	Type of arrhythmia			Treatment		
	Vitamin C group	Control group	*P*-value	Vitamin C group	Control group	*P*-value
Anesthesia induction	No arrhythmia: 23; PVC: 2	No arrhythmia: 25	0.49	No treatment: 25	No treatment: 25	0
End of bypass	No arrhythmia: 24; VF: 1	No arrhythmia: 24; VF: 1	0.99	No treatment: 24; lidocaine: 1	No treatment: 24; lidocaine: 1	0.99
6 h after surgery	No arrhythmia: 24; AF: 1	No arrhythmia: 22; AF: 1; PVC: 1	0.55	No treatment: 24; amiodarone: 1	No treatment: 22; amiodarone: 2; pacemaker: 1	0.49
24 h after surgery	No arrhythmia: 22; AF: 2; PVC: 1	No arrhythmia: 23; AF: 1; PVC: 1	0.83	No treatment: 24; amiodarone: 1	No treatment: 23; amiodarone: 2	0.50
After declamping	No arrhythmia: 18; AF: 2; PVC: 1; VF: 2	No arrhythmia: 16; AF: 3; V-tach: 2; AV-block: 2; VF: 2	0.30	No treatment: 18; lidocaine:4; lidocaine + shock: 3	No treatment: 16; lidocaine: 3; shock: 2; pacemaker: 1; lidocaine + shock: 3	0.66

AF=atrial fibrillation; AV-block=atrioventricular block; PVC=premature ventricular contraction; V-tach=ventricular tachycardia; VF=ventricular fibrillation

## DISCUSSION

In this study, the effect of high doses of vitamin C, intravenously and in cardioplegia solution, on the MI injury in 50 candidates for coronary artery bypass surgery were evaluated.

During ischemia, the disruption of the cell membrane integrity leads to release of calcium and phospholipid A2 and formation of polyunsaturated fatty acids and fatty acid radicals^[10]^. Thus, cells can release oxygen-derived free radicals and proteolytic enzymes, and these materials damage the cells containing deoxyribonucleic acid (DNA), proteins, and lipids^[11]^. Vitamin C has the ability of scavenging free radicals. This vitamin has cardioprotective properties, which were demonstrated to reduce oxidative damage in diabetic rats and during ischemia/reperfusion (I/R)-injury^[12,13]^. In some studies, vitamin C improved myocardial stunning and enhanced left ventricular function^[14]^. Also, vitamin C inhibits the expression of inducible nitric oxide synthetase (iNOS) in endothelial cells and neuronal nitric oxide synthetase (nNOS), and thereby reduces the level of NO in plasma, that is responsible for the guanylate cyclase activation, which counteracts the effects of vasoconstrictors. This might maintain the vascular resistance and baroreceptor reflex and, as a result, the mean arterial pressure could also be maintained^[15]^.

The focus of this study was to evaluate the effect of vitamin C on the plasma levels of CK-MB, troponin I, and LDH in four-time intervals (before anesthesia induction, at bypass termination, at six hours after surgery, and at 24 hours after surgery) in treatment and control groups. The results showed that the changes in the levels of all three cardiovascular biomedical markers over time in each group were significant within the group. CK-MB and troponin I changes were significant only between the first time and the three other times, indicating a significant increase in the cardiac biomarkers during and after the surgery. Also, in the LDH enzyme levels, the difference between the first time and the second time was relatively significant and the difference was quietly significant between the first and the third and fourth times. However, in comparing between the two groups, although the level of enzymes in the control group was always greater than in the treatment group, the difference was not significant between the groups. There was no significant difference between the two groups in each of the four study times. In the Oktar et al.^[16]^ study, the CK-MB level was lower in vitamin C group than in the control group. In another study, by Dingchao et al.^[5]^, the levels of CK-MB and LDH were higher in the control group after surgery than in the vitamin C group. In Lee et al.^[17]^ study, troponin I and LDH levels were lower in the treatment group than in the control group, but similarly to our study, the level of CK-MB was not significantly different. On the other hand, the outcome of this study is consistent with the study by Westhuyzen et al.^[18]^, where the pre-operation administration of supplements of vitamins C and E did not have a significant effect on myocardial damage and the level of CK-MB enzyme. Also, Gao et al.^[19]^ found out that the administration of 1 mM of vitamin C alone did not reduce post-ischemic injury, but coadministration with glutathione increased the protective effects of glutathione. All of these studies have different conditions, doses, and administration methods (intravenous, oral, mixed with a cardio-polycarbamide solution), and perhaps the reason for the different results is related to these variations.

In studies with a high dose of vitamin C^[5,16]^, the level of cardiac biomarkers was clearly reduced. In our study, at all times, with any reduction or increase process, the level of biomedical markers in the treatment group was lower than in the control group and this shows the clinical effect of vitamin C on reducing the levels of ischemic enzymes, but there was no statistically significant difference between the two groups, which may be due to a low sample size or a significant increase in biomedical markers at all intervals, especially from the first to the second time, which resulted in large dispersion of data and increased the SD, and the difference between the two groups was not significant. It is expected that by increasing the sample size or using longer intervals, the difference becomes statistically significant.

In this study, high doses of vitamin C were injected intravenously and in a cardioplegic solution in patients undergoing CABG surgery. According to the results, the significant decrease of all three biomarkers after the onset of the cardiopulmonary pump is clear.

The comparison between EF values showed an increase in EF in the control group within 72 hours after the operation, and this difference was significant. Also, the variable EF changes, which indicates the changes in EF before and after the operation, well demonstrates this difference. The percentage of EF in the vitamin C group showed 0.8 of increase and in the control group, it showed 0.4 of decrease. Therefore, it seems that vitamin C has been effective in improving the function of the left ventricle and the heart because patients receiving vitamin C have a better contractile power than those in the control group after 72 hours. This result is consistent with the study by Mak and Newton, which revealed that vitamin C has also enhanced the response of the left ventricle to the inotropic drugs, which improves left ventricular contraction^[20]^.

The length of ICU stay in patients receiving vitamin C was shorter than in those from the control group, and this difference was significant. Although the hospital stay and intubation time were shorter in the vitamin C group than in the control group, there were no significant differences between these two items in the two groups. This is consistent with the result of the study by Dinqchao et al.^[5]^, in which the patients had shorter ICU stay and hospital stay than the control group. Also, in Rezk et al.^[21]^ study, patients in the vitamin C group had shorter length of ICU stay than those in the control group.

There was no significant difference in the need for blood transfusion between the two groups. In fact, vitamin C seems to have no effect on reducing the need for blood in patients who have received it.

Regarding the need for inotrope support 24 hours after surgery in both groups and the number and hours of drug intake, vitamin C seems to have no effect on the need for inotropic drugs during the postoperative period. This conclusion is consistent with the study by Oktar et al.^[16]^.

The urinary output rate was measured before the onset of bypass until 24 hours postoperatively. The changes in both groups were similar over time, and the patients receiving vitamin C did not differ in the level of urinary output from those in the control group. The comparison of plasma levels of hemoglobin and potassium in the four periods, before the onset of the bypass until 24 hours after surgery, did not show any significant difference between the two groups. The changes made over time between the treatment and control groups had the same trend and vitamin C had no effect on this process.

Hemodynamic parameters, including systolic and diastolic blood pressures and heart rate, were compared in four-time intervals between patients from treatment and control groups. Blood pressure changes in both groups increased and decreased significantly, and no significant difference was observed. There was also a slight improvement in heart rate in the treatment group, but there was no statistically significant difference. Therefore, the vitamin C intervention did not have a clear effect on hemodynamic conditions in patients. These results are similar to the ones from Mak and Neutron study^[20]^. In another study by Jouybar et al.^[22]^, ascorbic acid had no significant effect on hemodynamic parameters, such as systolic and diastolic blood pressures and heart rate. But in the study by Lee et al.^[17]^, mean arterial blood pressure was better in the vitamin C group than in the control group, but the patients’ heart rate showed no difference between the two groups.

In this study, we monitored the incidence of arrhythmias before the onset of the bypass up to 24 hours after surgery in five intervals. Type and treatment of arrhythmias between the two groups did not differ significantly. The highest incidence of arrhythmias was observed at the time of aortic clamp removal, which was similar in the treatment and control groups. Compared to the control group, no significant changes were observed in the incidence of cardiac arrhythmias during and after operation. Vitamin C seems to have no effect on reducing the incidence of cardiac rhythm disturbances in patients. The result of this study is consistent with the ones from the study by Oktar et al.^[16]^. However, in the study by Rebrova et al.^[23]^, it was determined that vitamin C prevents cardiac rhythm disturbances 24 hours after surgery, that the use of vitamin C with beta blockers is recommended after bypass surgery to prevent arrhythmias, and it also suggested the use of vitamin C in patients with advanced heart disease. In the present study, patients have no acute underlying problems, such as severe cardiac arrhythmias, MI, very low EF, etc., and they were undergone surgery in a relatively stable general condition, and this may be a reason for ignoring the effect of vitamin C in preventing cardiac arrhythmias.

### Limitation

This study was performed on a limited number of patients and it is recommended to perform studies with larger sample sizes than this and in different countries, so the results could be applicable throughout the world.

## CONCLUSION

Vitamin C has significantly improved the ventricular function (EF) of patients 72 h after surgery and reduced the length of ICU stay.

No significant changes in cardiac biomarkers including CK-MB, troponin I, and LDH were seen over time in each group. For future researches, longer intervals (48 and 72 hours after surgery) or higher sample sizes are recommended. The combination of vitamin C with other antioxidants or their comparison can more accurately reveal its influence on prevention of coronary heart disease and reduction of cardiac biomarkers.

**Table:** 

Authors' roles & responsibilities	
NE	Substantial contributions to the conception or design of the work; or the acquisition, analysis, or interpretation of data for the work; final approval of the version to be published.
MHN	Drafting the work or revising it critically for important intellectual content; final approval of the version to be published.
MG	Agreement to be accountable for all aspects of the work in ensuring that questions related to the accuracy or integrity of any part of the work are appropriately investigated and resolved; final approval of the version to be published.
EA	Substantial contributions to the conception or design of the work; or the acquisition, analysis, or interpretation of data for the work; final approval of the version to be published.
